# Supplementary material for: Multi-Drug Cocktail Therapy Improves Survival and Neurological Function after Asphyxial Cardiac Arrest in Rodents
Source: Cells. 2023 Jun 5;12(11):1548. doi: 10.3390/cells12111548 (PMC10253071; doi:10.3390/cells12111548)
Supplement: Supplementary file 1 [file cells-12-01548-s001.zip › cells-2349967-supplementary.pdf]

cells

# Supplemental Methods

## Chemicals and Reagents

Drugs: Metformin (Sigma-Aldrich, USA), ATP-magnesium chloride (Sigma-Aldrich, USA), N-acetyl cysteine (Sigma-Aldrich, USA), poloxamer 188 (Sigma-Aldrich, USA), CoQ10 (Cayman Chemical, USA), cyclosporine A (Cayman Chemical, USA), Edaravone (Sigma-Aldrich, USA), SS-31 (Genemed Synthesis, USA), sulbutiamine (Toronto Research Chemical, Canada), vitamin C (Cayman Chemical, USA), and zoniporide (Toronto Research Chemical, Canada).

## Detailed Animal Experimental Procedure for 12 Minutes of Asphyxial CA

Adult male Sprague-Dawley rats (400-500 g) were obtained from the Charles River Laboratory (Wilmington, MA, USA) and housed in the animal center of the Feinstein Institutes for Medical Research on a 12-hour light/dark cycle with free access to water and food. Rats were anesthetized with 4% isoflurane (Isothesia, Butler-Schein AHS), intubated with a 14-gauge plastic catheter (Surflo, Terumo Medical Corporation, NJ, USA), and placed on mechanical ventilation under 2% isoflurane. The left femoral artery and vein were cannulated with polyethylene catheters (PE50, BD Intramedic, USA) to monitor mean arterial pressure and aid in drug infusion. After surgical preparation and heparin injection (300 IU), baseline physiologic parameters, such as MAP, HR, and end-tidal $CO_2$ ($ETCO_2$) were recorded using PowerLab and LabChart (ADInstruments, USA). The procedure for CA began with slowly injecting vecuronium bromide (2 mg/kg body weight; Hospira, USA) through the venous catheter over 4 min. Asphyxial CA was induced by switching off the ventilator and then discontinuing isoflurane. After 12 min of asphyxial CA, resuscitation was started: mechanical ventilation was resumed at 100% oxygen, and chest compressions were initiated. At the start of CPR, a bolus of epinephrine (20 μg/kg; International Medication System, Limited, USA) was injected through the venous catheter. CPR was continued until animals achieved ROSC, which was defined as MAP greater than 60 mmHg. If ROSC did not occur within 5 min after starting CPR, rats were excluded from the study. Hemodynamic recordings were continued until 2 h post-ROSC.

Blood samples were obtained from femoral artery at baseline, ROSC, and 20, 40, and 120 min post-ROSC. To analyze arterial blood gas, we measured pH, $pCO_2$, $pO_2$, bicarbonate, lactate, and $SaO_2$ saturation at baseline, and 20 and 40 min post-ROSC. Blood glucose was measured at baseline, ROSC, and 20, 40, and 120 min post-ROSC. Then, 120 min post-ROSC, animals were extubated, decannulated, sutured, and provided postoperative care, which included subcutaneous saline and Buprenex (buprenorphine; 0.03 mg/kg body weight; Par Pharmaceutical, USA). Animals were returned to the animal housing facility and provided daily care according to the approved protocol. Animals were monitored for 72 h survival, and neurological function was evaluated at 24, 48, and 72 h post-ROSC by a blinded researcher using 2 scoring scales. Then rats were euthanized, and whole brain was harvested for histological analysis of neuronal degeneration.

## Detailed Animal Experimental Procedure for 20 Min of Asphyxial CA and 30 Min of Cardiopulmonary Bypass Resuscitation (CPB)

Adult male Sprague-Dawley rats (400-500 g) were used for this study. After anesthetization rats were placed on mechanical ventilation under 2% isoflurane. The left femoral artery and vein were cannulated with polyethylene catheters (PE50, BD Intramedic, USA) to monitor mean arterial pressure and aid in drug infusion. The right jugular vein and the right femoral artery were cannulated for venous outflow and the arterial inflow of CPB fluid. After surgical preparation and heparin injection (300 IU), baseline physiologic parameters, such as MAP, HR, and end-tidal $CO_2$ ($ETCO_2$) were recorded using PowerLab and LabChart (ADInstruments, USA). The procedure for CA began with slowly injecting

vecuronium bromide (2 mg/kg body weight; Hospira, USA) through the venous catheter over 4 min. After waiting 3 more min asphyxial CA was induced by switching off the ventilator and then discontinuing isoflurane. After 20 min of asphyxial CA, resuscitation was started, with the initiation of CPB flow and resumption of ventilation. The customized CPB circuit designed for rodents consists of a heat exchanger, an open venous reservoir, a membrane oxygenator, and a roller pump. For the oxygenator, 100% oxygen was used to saturate blood with oxygen. The open venous reservoir was filled with a 20 mL solution containing Normosol-R Electrolyte Injection Solution (10 mL), 6% hetastarch (10 mL), and 8.4% sodium bicarbonate (0.3 mL). The initial CPB flow rate was 20 mL/min and incresed to 70 mL/min, then gradually decreased to meet venous outflow. During this, the complement solution containing plasmalyte A (2.5 mL), 6% hetastarch (2.5 mL) and 10 mL blood from a donor rat was added to the reservoir as needed. Immediately after achieving ROSC, vehicle/cocktail were injected through the femoral vein over 20 min using infusion pump. After 30 min of initiation of CPB, the bypass machine was stopped. We used a syringe pump to continuously infuse 20 mL supplement solution containing 17 mL of Normosol-R Electrolyte Injection Solution, 3 mL of 50% dextrose and 8.4% sodium bicarbonate (0.5 mL), as per the animal's body weight in mL/hour. Ventilation was subsequently adjusted to a $PaCO_2$ of 35 to 45 mm Hg. Initially, animals were ventilated with 100% oxygen. The oxygen concentration was decreased to 60% after 60 min.

# Data Supplement

**Table S1.** Cocktail Development and Trials to Assess Survival Benefit.

| Drug formulation number | Drugs included | Cocktail formulation | Study details | 72 h survival |
|---|---|---|---|---|
| NWH-1 | ATP-$MgCl_2$, N-acetyl cysteine, sulbutiamine, zoniporide, cyclosporine A, CoQ10, vitamin C, and edaravone | CoQ10, cyclosporine A, and sulbutiamine were insoluble in water | Measured effects of each drug on hemodynamics and survival without inducing CA | Yes |
| NWH-2 | ATP-$MgCl_2$, N-acetyl cysteine, sulbutiamine, zoniporide, cyclosporine A, CoQ10, vitamin C, and edaravone | The formulation developed, but pH was not adjusted | Monitored hemodynamics and survival | Yes |
| NWH-3 | ATP-$MgCl_2$, N-acetyl cysteine, poloxamer 188, SS-31, sulbutiamine, zoniporide, cyclosporine A, CoQ10, vitamin C, and edaravone | Drugs solubilized in 1X PBS, and pH was adjusted with sodium bicarbonate. | Monitored hemodynamics and survival after 12 min CA | Non-CA rats survived; CA rats died |
| NWH-4 | ATP-$MgCl_2$, N-acetyl cysteine, poloxamer 188, SS-31, sulbutiamine, zoniporide, cyclosporine A, CoQ10, vitamin C, and edaravone | Cyclosporine A dissolved in 1X PBS, and pH adjusted with sodium bicarbonate. | Monitored survival after 12 min CA | No |
| NWH-5 | ATP-$MgCl_2$, N-acetyl cysteine, poloxamer 188, SS-31, sulbutiamine, zoniporide, cyclosporine A, CoQ10, vitamin C, and edaravone | ATP-MgCl2 and zoniporide separately injected. Drugs solubilized in 1X PBS,and pH adjusted with sodium bicarbonate. | Monitored survival after 12 min CA | 1 CA rat survived; 3 CA rats died |
| NWH-6 | Metformin, ATP-$MgCl_2$, N-acetyl cysteine, poloxamer 188, SS-31, sulbutiamine, zoniporide, cyclosporine A, CoQ10, vitamin C, and edaravone | ATP-MgCl2 separately injected. Drugs solubilized in 1X PBS, and pH adjusted with sodium bicarbonate. Metformin replaced sodium amobarbital. | Monitored survival after 12 min CA | No |

| | | | | |
|---|---|---|---|---|
| NWH-7 | Metformin, ATP-$MgCl_2$, N-acetyl cysteine, poloxamer 188, SS-31, sulbutiamine, zoniporide, cyclosporine A, CoQ10, vitamin C, edaravone, and hydrogen gas | ATP-$MgCl_2$ separately injected. Drugs solubilized in 1X PBS, and pH adjusted with sodium bicarbonate. Hydrogen gas added. | Monitored survival after 10 min and 12 min CA | 1 10 min CA rat survived; 12 min CA rats died |
| NWH-8 | Metformin, ATP-$MgCl_2$, N-acetyl cysteine, poloxamer 188, SS-31, sulbutiamine, zoniporide, cyclosporine A, CoQ10, vitamin C, edaravone, and hydrogen gas | ATP-$MgCl_2$ removed. Drugs solubilized in 1X PBS, and pH adjusted with sodium bicarbonate. Hydrogen gas added. | Monitored survival after 10 min CA | 1 CA rat survived; 7 CA rats died |
| NWH-9 | Metformin, ATP-$MgCl_2$, N-acetyl cysteine, poloxamer 188, SS-31, sulbutiamine, zoniporide, cyclosporine A, CoQ10, vitamin C, and edaravone | ATP-$MgCl_2$ and hydrogen gas removed. Drugs solubilized in 1X PBS, and pH adjusted with sodium bicarbonate. | Monitored survival after 10 min and 12 min CA | All 3 10 min CA rats survived; all 5 12 min CA rats survived after cocktail treatment; all 5 12 min CA rats died after vehicle treatment |

Abbreviations: ATP-$MgCl_2$ indicates adenosine triphosphate-magnesium chloride; PBS indicates phosphate-buffered saline.

**Table S2.** Scale 1—Modified Neurological Deficit Score for Rats *.

| **Parameter Characteristic Score** | | | |
|---|---|---|---|
| **General: 200 points** | | | |
| Consciousness | Unresponsive=0 | Depressed=50 | Normal=100 |
| Respiration, breaths per min | (<60, >120)=100 | (<121, >140)=50 | (>141)=0 |
| **Cranial nerves: 100 points** | | | |
| Olfactory orient to smell | No=0 | Yes=20 | |
| Startle response to visual stimulus | No=0 | Yes=20 | |
| Cornel reflex blink response to corneal stimulus | No=0 | Yes=20 | |
| Whisker movement, spontaneous | No=0 | Yes=20 | |
| Hearing—startle response to loud noise | No=0 | Yes=20 | |
| **Motor: 50 points** | | | |
| Left forepaw—spontaneous or withdraw from pain | No=0 | Yes=10 | |
| Right forepaw—spontaneous or withdraw from pain | No=0 | Yes=10 | |
| Left hind paw—spontaneous or withdraw from pain | No=0 | Yes=10 | |
| Right hind paw—spontaneous or withdraw from pain | No=0 | Yes=10 | |
| Tail—spontaneous or withdraw from pain | No=0 | Yes=10 | |
| **Sensory: 50 points** | | | |
| Left forepaw—react to pain | No=0 | Yes=10 | |
| Right forepaw—react to pain | No=0 | Yes=10 | |
| Left hind paw—react to pain | No=0 | Yes=10 | |
| Right hind paw—react to pain | No=0 | Yes=10 | |
| Tail—react to pain | No=0 | Yes=10 | |
| **Coordination: 100 points** | | | |
| Ledge traverse | No=0 | Yes=25 | |

| | | |
|---|---|---|
| Righting reflex | No=0 | Yes=25 |
| Placing test | No=0 | Yes=25 |
| Stop at table edge | No=0 | Yes=25 |
| **Total score** | | |

* Adapted from Neumar *et al.*, 1995.

**Table S3.** Scale 2—Neurological Deficit Score*.

| **General Behavioral Deficit** | **Total Score: 19** |
|---|---|
| Consciousness | Normal=10<br>Stuporous=5<br>Comatose or unresponsive=0 |
| Arousal | Eyes open spontaneously=3<br>Eyes open to pain=1<br>No eye-opening=0 |
| Respiration | Normal=6<br>Abnormal (hypoventilation or hyperventilation)=3<br>Absent=0 |
| **Brain stem function** | **Total score: 21** |
| Olfaction—response to smell of food | Present=3<br>Absent=0 |
| Vision—head movement to light | Present=3<br>Absent=0 |
| Pupillary reflex—pupillary light reflex | Present=3<br>Absent=0 |
| Corneal reflex | Present=3<br>Absent=0 |
| Startle reflex | Present=3<br>Absent=0 |
| Whisker stimulation | Present=3<br>Absent=0 |
| Swallowing, liquids or solids | Present=3<br>Absent=0 |
| **Motor assessment—strength (test left and right separately)** | **Total score: 6** |
| | Normal=3<br>Stiff or weak=1<br>No movement or paralyzed=0 |
| **Sensory assessment—pain (test left and right separately** | **Total score: 6** |
| | Brisk withdrawal with pain=3<br>Weak or abnormal response (extension or flexion posture)=1<br>No withdrawal=0 |
| **Motor behavior** | **Total score: 6** |
| Gait coordination | Normal=3<br>Abnormal=1<br>Absent=0 |
| Balance beam walking | Normal=3<br>Abnormal=1<br>Absent=0 |
| **Behavior** | **Total score: 12** |
| Righting reflex | Normal=3<br>Abnormal=1<br>Absent=0 |

| | |
|---|---|
| Negative geotaxis | Normal=3<br>Abnormal=1<br>Absent=0 |
| Visual placing | Normal=3<br>Abnormal=1<br>Absent=0 |
| Turning alley | Normal=3<br>Abnormal=1<br>Absent=0 |
| **Seizures** | **Total score: 10** |
| Convulsive or non-convulsive | No seizure=10<br>Focal seizure=5<br>General seizure=0 |

* Described in Jia et al. 2008.

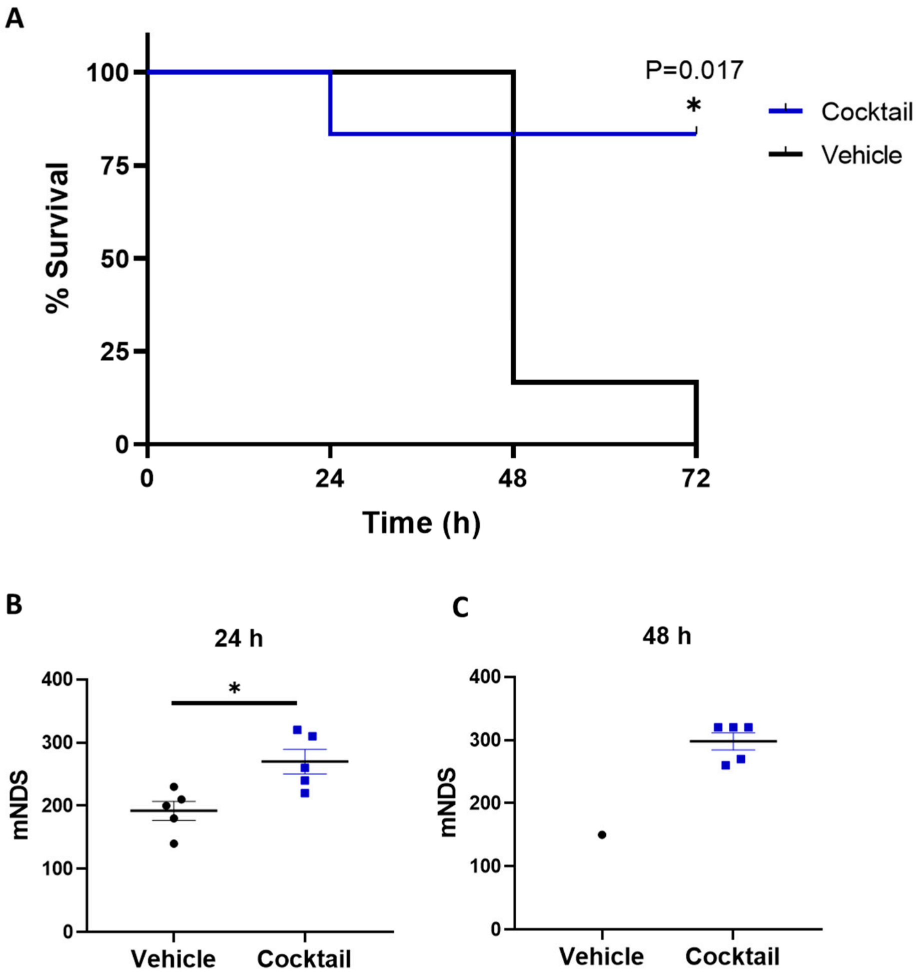


**Figure S1.** Preliminary study of survival and neurological deficit in cocktail-treated and vehicle-treated rats after cardiac arrest. (A) Survival analysis of cocktail-treated and vehicle-treated rats after 12 min cardiac arrest (CA) and resuscitation in the preliminary study. Survival was substantially greater in cocktail-treated rats at 72 h post-ROSC. (B,C) Modified neurological deficit score (mNDS) of vehicle-treated and cocktail-treated rats after 12 min CA at (B) 24 and (C) 48 h post-resuscitation in the preliminary study. Rats who did not survive (score = 0) were excluded from the statistical analysis. Scores in cocktail-treated rats were significantly higher than vehicle-treated rats at 24 h after return of spontaneous circulation (ROSC). Only 1 vehicle-treated rat survived at 48 h post-ROSC. Data are presented as mean ± SEM. * $p < 0.05$ between vehicle and cocktail.

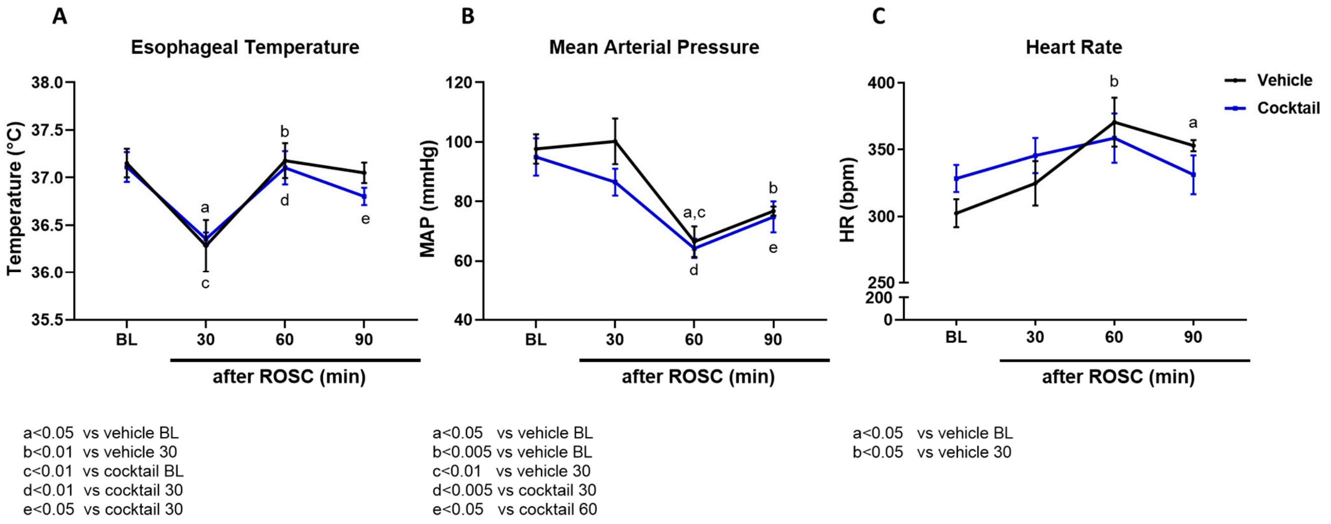


**Figure S2.** Preliminary study of esophageal temperature and hemodynamic changes in cocktail-treated and vehicle-treated rats after cardiac arrest. (A) through (C), Changes in (A) esophageal temperature, (B) mean arterial pressure (MAP), and (C) heart rate (HR) in bpm (beats per minute) at baseline (BL) and after return of spontaneous circulation (ROSC) in preliminary 12 min CA study. No major differences occurred between vehicle-treated and cocktail-treated rats. Data are presented as mean ± SEM, and significance comparisons intragroup are shown in the inserts.

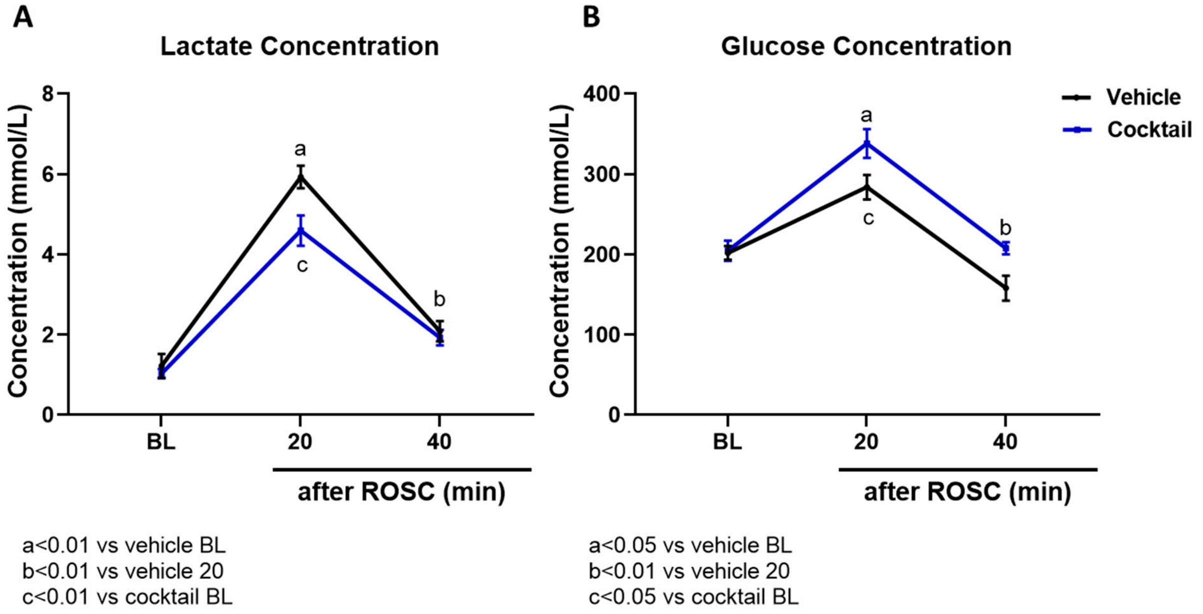


**Figure S3.** Preliminary study of lactate and glucose concentration in cocktail-treated and vehicle-treated rats after cardiac arrest. (A,B), Changes in (A) blood lactate and (B) glucose concentrations at baseline (BL) and after return of spontaneous circulation (ROSC) in preliminary 12 min CA study. No major differences occurred between vehicle-treated and cocktail-treated rats. Data are presented as mean ± SEM, and significance comparisons intragroup are shown in the inserts.

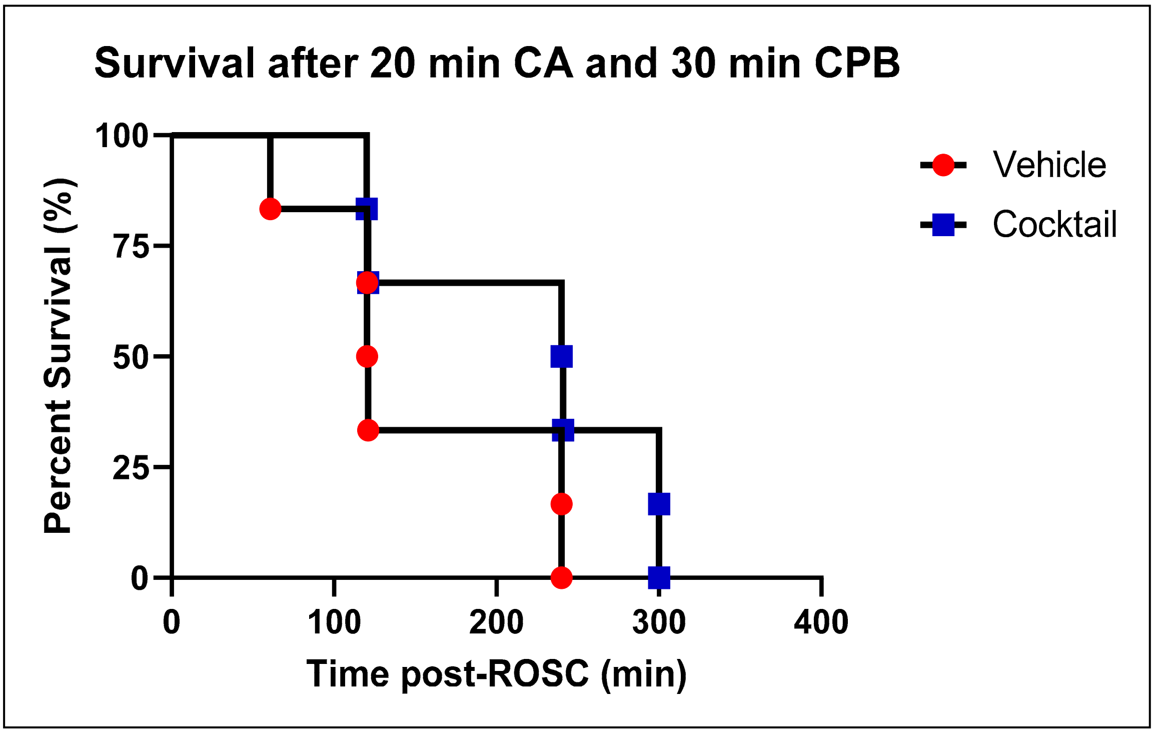


**Figure S4. Preliminary study of survival in cocktail-treated and vehicle-treated rats after highly lethal cardiac arrest.** Survival analysis of cocktail-treated and vehicle-treated rats after 20 min cardiac arrest (CA) and 30 min cardiopulmonary bypass (CPB) resuscitation in the preliminary study. Cocktail-treated rats tended to have increased survival times compared to vehicle-treated rats.
